# Global implementation of Good Participatory Practice Guidelines for biomedical HIV prevention research: charting progress and setting milestones

**DOI:** 10.1186/1742-4690-9-S2-P240

**Published:** 2012-09-13

**Authors:** S Hannah, M Warren, E Bass

**Affiliations:** 1 AVAC, New York, NY, USA

## Background

In 2007, UNAIDS and AVAC developed the Good Participatory Practice (GPP) Guidelines with the aim of ensuring community and stakeholder engagement in HIV prevention research. The second edition, launched in 2011, provides the first standardized framework for GPP compliance. Five years after initial publication, it is possible and necessary to evaluate milestones in adoption of the guidelines at trial site, sponsor, country, and international levels in order to gauge progress and next steps.

## Methods

A systematic review of 1) articles citing or mentioning GPP; 2) GPP-specific trainings requested and/or carried out by AVAC partners; 3) national and international forums in which GPP was mentioned, adopted or presented shows that GPP is gaining currency and relevance as key in guiding not only prevention research but all research involving human subjects.

## Results

Support for and awareness of GPP have increased dramatically over the past five years. There is, however, a continued need for technical support for implementation. Common gaps in practice included low levels of documentation of engagement activities, planning around possible contentious issues and trial results, and stakeholder input in trial protocols. National level implementation activities provided initial steps in requirement of GPP. For example, GPP was presented to US President Obama’s Commission for Bioethics, and subsequently cited as recommendation for community engagement in the Commission’s official report. This effort secured international recognition and expanded relevance beyond HIV prevention research. Additional action is necessary for requirement and monitoring of practices.

## Conclusion

While stakeholder engagement has long been accepted practice in HIV prevention research, findings from multiple implementation processes suggest the value and power of applying a systematic framework to practices. Awareness of GPP has increased, however meaningful implementation within and outside of HIV prevention research necessitates sustained commitments, new resources and action on the part of national, international and community-level stakeholders.

